# Effects of transcranial direct current stimulation in children and young people with psychiatric disorders: a systematic review

**DOI:** 10.1007/s00787-023-02157-0

**Published:** 2023-02-11

**Authors:** Lucy Gallop, Samuel J. Westwood, Yael Lewis, Iain C. Campbell, Ulrike Schmidt

**Affiliations:** 1 Section of Eating Disorders, Department of Psychological Medicine, Institute of Psychiatry, Psychology & Neuroscience, King’s College London, De Crespigny Park, PO Box 59, London, SE5 8AF UK; 2 Department of Psychology, School of Social Science, University of Westminster, London, W1W 6UW UK; 3 Institute of Psychiatry, Psychology and Neuroscience, King’s College London, London, SE5 8AB UK; 4 Hadarim Eating Disorder Unit, Shalvata Mental Health Centre, Hod Hasharon, Israel; 5 Sackler Faculty of Medicine, Tel-Aviv University, Tel-Aviv, Israel; 6 South London and Maudsley NHS Foundation Trust, London, UK

**Keywords:** tDCS, Children, Young people, Psychiatric disorders, Systematic review

## Abstract

Transcranial direct current stimulation (tDCS) has demonstrated benefits in adults with various psychiatric disorders, but its clinical utility in children and young people (CYP) remains unclear. This PRISMA systematic review used published and ongoing studies to examine the effects of tDCS on disorder-specific symptoms, mood and neurocognition in CYP with psychiatric disorders. We searched Medline via PubMed, Embase, PsychINFO via OVID, and Clinicaltrials.gov up to December 2022. Eligible studies involved multiple session (i.e., treatment) tDCS in CYP (≤ 25 years old) with psychiatric disorders. Two independent raters assessed the eligibility of studies and extracted data using a custom-built form. Of 33 eligible studies (participant *N* = 517), the majority (*n* = 27) reported an improvement in at least one outcome measure of disorder-specific symptoms. Few studies (*n* = 13) examined tDCS effects on mood and/or neurocognition, but findings were mainly positive. Overall, tDCS was well tolerated with minimal side effects. Of 11 eligible ongoing studies, many are sham-controlled RCTs (*n* = 9) with better blinding techniques and a larger estimated participant enrolment (*M* = 79.7; range 15–172) than published studies. Although encouraging, the evidence to date is insufficient to firmly conclude that tDCS can improve clinical symptoms, mood, or cognition in CYP with psychiatric disorders. Ongoing studies appear of improved methodological quality; however, future studies should broaden outcome measures to more comprehensively assess the effects of tDCS and develop dosage guidance (i.e., treatment regimens).

**Supplementary Information:**

The online version contains supplementary material available at 10.1007/s00787-023-02157-0.

## Introduction

It is estimated that 10–20% of children and adolescents experience mental health disorders worldwide [1], with roughly 75% of all psychiatric disorders having an onset in childhood, adolescence, or early adulthood (mid-20 s) [2, 3]. This period of onset coincides with sensitive periods of experience-dependent changes in brain structure and function, with evidence showing common and disorder-specific functional disorganisation of neurocognitive and affective networks in children and young people (CYP) with psychiatric disorders (e.g. [4].). This highlights the need for early detection and intervention [5]; however, pharmacotherapy in CYP remains contentious across many mental health conditions [6, 7] and the widespread imbalance between demand and capacity in child and adolescent mental health services may limit CYP from accessing timely, quality mental health care [8].

Neuromodulation techniques, particularly non-invasive brain stimulation, are safe and promising treatment alternatives and/or adjuncts that could be used to bridge the mental health treatment gap [9]. One particular technique, transcranial direct current stimulation (tDCS), involves the application of low amplitude (e.g., 1–2 mA), sustained current over a short duration (e.g., 20 min) via strategically positioned electrodes on the scalp [10]. From a mechanistic perspective, the short- and longer-term effects of tDCS on cortical excitability are polarity-specific, i.e., anodal tDCS increases excitability of local neurons and cathodal TDCS decreases excitability [11]. The immediate effects of tDCS relate to a shift in resting transmembrane potential of the neurons stimulated [12], whereas post-stimulation effects are proposed to rely on N-methyl-D-aspartate (NMDA) glutamate receptor-dependent neuroplastic changes, similar to those occurring in long-term potentiation (LTP) and depression (LTD) [13]. Evidence has also shown that excitatory after-effects of anodal tDCS are mediated by a reduction in intracortical gamma aminobutyric acid (GABA), whereas cathodal tDCS after-effects are mediated by reduced glutamate concentrations [14–16]. Modulation of neural activity in regions under the stimulating electrode [17], as well as in distal, interconnected regions [18], makes tDCS ideal for use in neuropsychiatric disorders associated with hyper- or hypo- cerebral excitability patterns.

The duration and strength of tDCS after-effects on neuronal excitability and/or synaptic strength is contingent upon the stimulation parameters used (e.g., intensity, duration, location, and number of sessions) (e.g., [19]), as is its safety and tolerability. A comprehensive review [20] reported no serious/irreversible adverse events in over > 33,000 sessions of tDCS applied at < 4 mA, for < 40 min, and a total charge of < 7.2 Coulombs. A recent systematic review [21] of safety and tolerability in 156 CYP found that 864 tDCS sessions applied within the standards (i.e., 0–2 mA, 10–20 min, 1–20 sessions) produced no serious/irreversible adverse events and reported side-effects were similar to those in adults, with tingling (25–58%) and itching (25–54%) the most frequently reported. This, in combination with its portability and relatively low cost, makes tDCS a promising therapeutic tool for CYP with psychiatric disorders.

In psychiatric research, tDCS has been mainly administered to adults. The data have resulted in meta-analytic evidence and promising reviews of beneficial effects (e.g. [22, 23]). Studies involving CYP are growing rapidly, with > 20 studies published or registered as trials on clinicaltrials.gov in the past 2-years (see also, [24, 25]). Despite this, attempts to consolidate findings in CYP are outdated or limited to non-systematic, narrative reviews [26, 27] or systematic reviews that have focused on literature for a specific disorder, particularly neurodevelopmental disorders [28, 29], or the safety and tolerability of tDCS [21]. None have examined tDCS-effects on mood or cognition in CYP with psychiatric disorders. Therefore, we followed a rigorous methodology for systematic reviews focusing on published and unpublished studies in CYP with psychiatric disorders.

Following PRISMA 2020 (Preferred Reporting Items for Systematic Reviews and Meta-Analyses) guidelines [30], we systematically reviewed studies investigating tDCS effects across psychiatric disorders in CYP in order to (1) evaluate the effects of tDCS on disorder-specific symptoms and impairments, (2) determine the effects of tDCS on mood and neurocognitive outcomes, and (3) outline the populations and methodologies used in ongoing trials and unpublished data.

## Methods

### Protocol and registration

This study was pre-registered (see PROSPERO, ID: CRD42019158957; and [31]) and is reported in line with PRISMA 2020 guidelines [30].

### Literature search

MEDLINE, EMBASE and PsycINFO databases were searched using the following search terms: (transcranial direct current stimulation or tDCS) AND (young people, child, adolescent, young adult, youth, boy, girl, paediatric, young people and young persons) AND (neuropsychiatric disorders, autism, ADHD, schizophrenia, mood disorder, bipolar, depression, anxiety, panic, OCD, Tourette’s, PTSD, acute stress disorder, substance abuse and eating disorders, personality disorder). The search was conducted on 28/01/21 and was updated on 26/01/22 and 09/12/22. The reference lists of included studies were manually searched for additional relevant studies not identified by the database search. To identify ongoing/unpublished trials, we searched World Health Organisation International Clinical Trials Registry Platform (ICTRP) registry, the National Institute of Health (NIH) registry, the European Union Clinical Trials Register, and the International Standard Randomised Controlled Trials Number (ISRCTN) registry.

### Eligibility criteria

We included all types of full-text publications written in English that reported multiple (> 1) sessions of tDCS in individuals under 26 years of age at enrolment with a psychiatric disorder. We included all types of reports, studies and multi-session tDCS protocols unless the aim was basic research, protocol development, or to investigate the mechanism of action of tDCS.

### Data extraction and analysis

Two authors (LG and YL) independently screened identified records against the eligibility criteria, extracted data, and performed the quality assessment. Data extraction was performed with a custom-made form adapted from the Cochrane data collection for intervention reviews [32] (see Supplementary Material S1 for details). A meta-analysis was not feasible due to significant heterogeneity in study designs, outcome measures, and tDCS protocols.

### Quality assessment

LG and YL independently assessed risk of bias using the Cochrane risk of bias 2.0 tool (RoB 2.0) in randomised controlled trials (RCTs) [33] and the Cochrane tool for risk of bias in non-randomised studies of interventions (ROBINS-I) [34]. Inter-rater agreement was 92%. Conflicts were resolved by discussion.

## Results

**Fig. 1:**
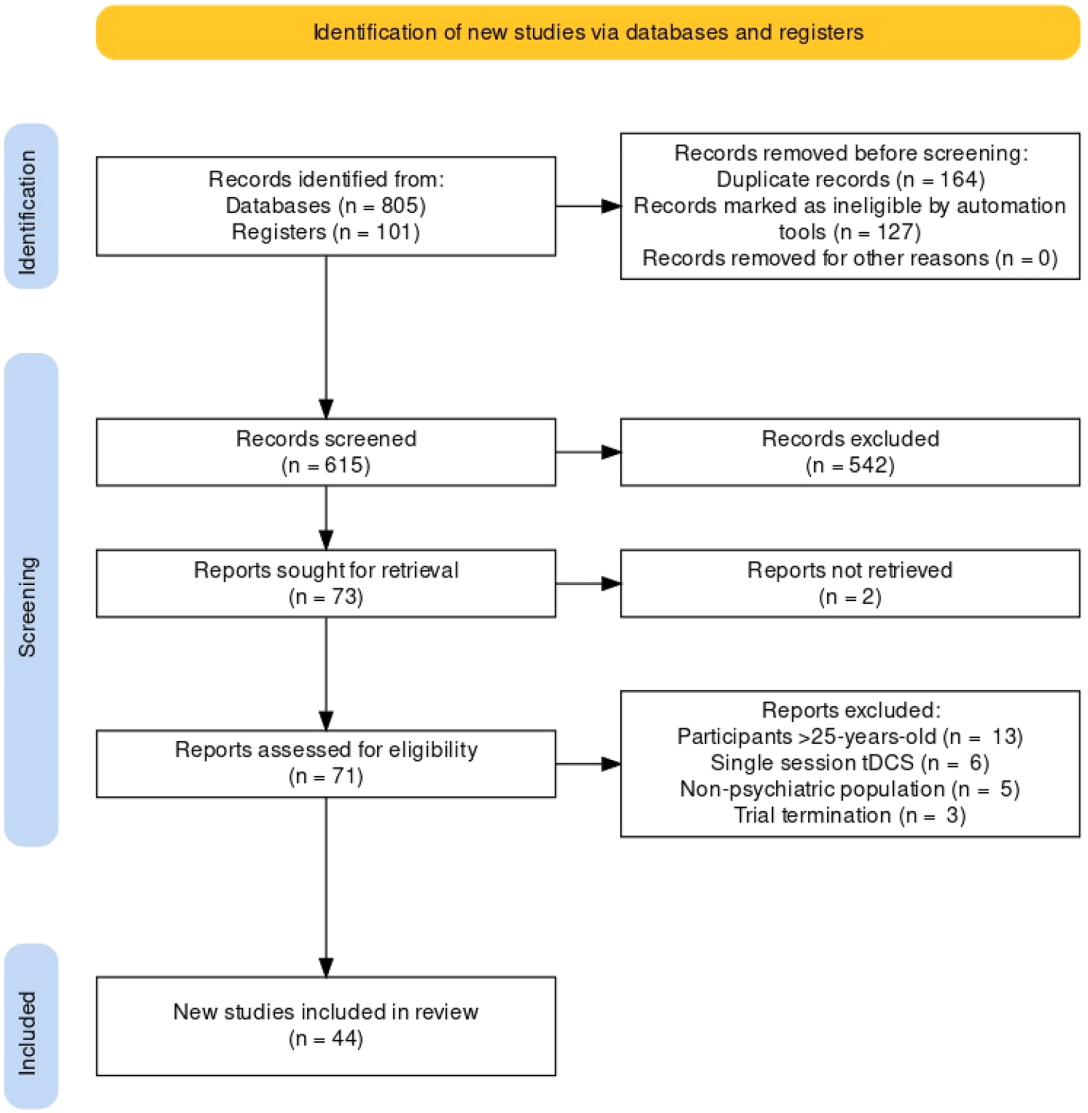
PRISMA flow diagram of selected studies (*n* = number of articles). A total of 33 studies were systematically reviewed and 11 ongoing/unpublished trials were identified

We identified 33 eligible studies (total *N* = 517; age range 2–25 years, *M* = 15.53, SD = 6.44; 82.2% male), composed of eight double-blind RCTs [37–40, 43, 44, 46, 48], four double-blind, crossover RCTs [35, 43, 45, 47], one double-blind, sham-controlled trial [67], two single-blind RCTs [41, 63], one single-blind controlled clinical trial [65], four open-label studies [36, 38, 44, 62], and 14 case series/studies [42, 51–61, 64, 66] (see Table 1 and 2). Seven studies were in ASD [35–43], seven in ADHD [44–50], four in schizophrenia [51–54], two in OCD [55, 56], Tourette’s syndrome [57, 58], depression [59, 60], anxiety [61, 62], or substance abuse disorder [63, 64], and one each in eating disorders [65], catatonia [66], or co-comorbid ASD, ADHD and anxiety [67]. Across studies, tDCS was typically delivered for 20 to 30 min (*M* = 21.61; SD = 4.38) once-daily over 4 to 28 sessions (*M* = 10.75; SD = 6.15). However, several studies applied tDCS in twice-daily sessions [51, 53–57, 67], one of which suggested that tDCS remains an ongoing treatment [51]. Montage configurations included anodal (*n* = 15)*,* cathodal *(n* = 3)*,* and bilateral (*n* = 15) tDCS, with anodal tDCS to the left-DLPFC (*n* = 10) and bilateral tDCS to the DLPFC (*n* = 6) the most frequently used across studies. tDCS was delivered at a stimulation intensity between 0.25 and 3 mA, with 1 mA (*n* = 13) or 2 mA (*n* = 11) most employed (Fig. 1).

**Table 1:** Summary of controlled trials using transcranial direct current stimulation in children, adolescents, and young people with psychiatric disorders

Authors (year)	Design	Control group	*N*	Diagnosis	Age: mean (range)	Anode/cathode	tDCS Protocol	Disorder-specific outcome measures	RoB	Reported AEs
Auvichayapat et al., (2022) [37]	Double-blind, multi-arm RCT	Sham-tDCS	36	ASD	6.2 (3–7)	L-DLPFC/R-shoulder	5 or 20 sessions; 1 mA; n/r	*CARS; ATEC total; ***ATEC-Social***; ATEC-Sensory; ATEC-Behaviour; ATEC-Speech*	**–**	5- vs. 20-sessions tDCS vs. sham tDCS: Irritability (8% vs. 8% vs. 0%); Insomnia (8% vs. 0% vs. 0%)
Han et al., (2022) [40]	Double-blind RCT	Sham-tDCS + CRT	41	ASD	17.6 (14–21)	R-SOA/L-DLPFC	10 sessions; 1.5 mA; 20 min	*SRS-2; ***SCI***; RRB*	**X**	Active vs. sham tDCS group^(a)^: Itching (50% vs. 19%, ***p* < 0.05**); Headache (5% vs. 0%); Scalp pain (5% vs. 0%); Tingling (25% vs. 5%); Sleeping problem (10% vs. 0%); Trouble concentrating (10% vs. 5%)
Zemestani et al., (2021) [43]	Double-blind RCT	Sham-tDCS	32	ASD	8.1 (7–12)	L-DLPFC/R-DLPFC	10 sessions; 1.5 mA; 15 min	*GARS-Stereotypical; ***GARS-Communication; GARS-Sociability; GARS-Total; ToM-1***; ToM-2; ToM-3; ToM-Total; ***CPRS-RS***; ERC*	**X**	Not measured or reported
Qiu et al., (2021) [41]	Single-blind RCT	Sham-tDCS	40	ASD	4.4 (2–6)	L-DLPFC/R-shoulder	15 sessions; 1 mA; 20 min	**ABC; RBS-R; CARS***; CSHQ; CARS-GI*	**X**	One parent report of hyperactivity after 5 real tDCS sessions which remitted 1-week after final tDCS session
Hadoush et al., (2020) [39]	Double-blind RCT	Sham-tDCS	43	ASD	7.8 (4–14)	L-DLPFC & R-DLPFC/L-SOA & R-SOA	10 sessions; 1 mA; 20 min	*ATEC total; ***ATEC-Speech***; ATEC-Social; ***ATEC-Sensory***; ATEC-Behaviour*	**X**	Reported there were ‘no AEs’
Amatachaya et al., (2014) [35]	Double-blind, crossover RCT	Sham-tDCS	20	ASD	6.4 (5–8)	L-DLPFC/R**-**shoulder	5 sessions; 1 mA; 20 min	*ATEC total; ***ATEC-Speech***; ATEC-Social; ATEC-Sensory; ATEC-Behaviour; CARS; CGAS; CGI-I*	**–**	Reported there were ‘no AEs’
Schertz et al., (2022) [48]	Double-blind RCT	Sham-tDCS + CT	25	ADHD	10.8 (8–16)	L-DLPFC/Vertex	12 sessions; 1 mA; 20 min	**VADPRS; CBCL**	**X**	Active tDCS vs. sham tDCS^(b)^: Burning sensation (0.20 vs. 0.27, ***p* < 0.05**); Dizziness (0.03 vs. 0); Headache (0.05 vs. 0.09, ***p* < 0.05**); Itching sensation (0.30 vs. 0.17, ***p* < 0.05**); Neck pain (0 vs. 0.01); Scalp pain (0.11 vs. 0.14, ***p* < 0.05**); Skin redness (0.09 vs. 0.01); Sleepiness (0.08 vs. 0.02); Tingling sensation (0.30 vs. 0.39, ***p* < 0.05**); Trouble concentrating (0.02 vs. − 0.01)
Klomjai et al., (2022) [47]	Double-blind, crossover RCT	Sham-tDCS	11	ADHD	8.6 (7–14)	R-SOA/L-DLPFC	5 sessions; 1.5 mA; 20 min	Only cognitive outcomes	**–**	Not measured or reported
Berger et al., (2021) [45]	Double-blind, crossover RCT	tRNS	19	ADHD	9.4 (6–12)	L-DLPFC/R-SOA	5 sessions; 0.75 mA; 20 min	**ADHD-RS**	**X**	Active tDCS vs. active tRNS group: Itching (32% vs. 21%); Headache (11% vs. 0%); Local redness (5% vs. 0%); Tingling (11% vs. 11%); Discomfort (11% vs. 0%)
Breitling-Ziegler et al., (2021) [46]	Double-blind RCT	Sham-tDCS	33	ADHD	13 (10–17)	R-IFG/R-IFG	5 sessions; 0.5 or 0.25 mA; 20 min	**DISYPS-II (self- and parent-rated)**	**–**	0.5 mA vs. 0.25 mA vs. sham-tDCS^(a)^: Itching (33% vs. 64% vs. 23%); Headache (22% vs. 45% vs. 39%); Painful sensation (33% vs. 64% vs. 23%); Burning sensation (11% vs. 9% vs. 8%); Vertigo (11% vs. 9% vs. 8%); Nausea (0% vs. 0% vs. 8%); Fatigue (33% vs. 46% vs. 46%); Insomnia (11% vs. 9% vs. 15%); Phosphenes (0% vs. 9% vs. 8%)
Westwood et al., (2020) [50]	Double-blind RCT	Sham-tDCS	50	ADHD	13.6 (10–18)	R-IFC/R-SOA	15 sessions; 1 mA; 20 min	**ADHD-RS; Conners 3-P; WREMB-R; CIS; ARI; MEWS**	**–**	Active tDCS vs. sham tDCS group^(a)^: Side effects (14.6 vs. 11.9); Adverse effects (16.3 vs. 15, ***p* < 0.05**)
Soff et al., (2017) [49]	Double-blind, crossover RCT	Sham-tDCS	15	ADHD	14.2 (12–16)	L-DLPFC/Vertex	5 sessions; 1 mA; 20 min	*FBB-Inattention (1-week FU)*;** FBB-Hyperactivity; FBB-Impulsivity**	**–**	Active tDCS vs. sham tDCS^(a)^: Tingling/itching sensation (46% vs. 46%); Headache (13% vs. 0%)
Francis et al., (2020) [67]	Double-blind, controlled trial	Sham-tDCS	2	ASD, ADHD, and anxiety	15	R-IFG/L-OFC	10 sessions; 1 mA; 13 min	**CY-BOCS; ***OCI; RBS-R***; ADHD-RS**	**X**	Active tDCS vs. sham tDCS: Itching (YES vs. NO); Irritability (YES vs. NO); Neck pain (YES vs. NO)
Costanzo et al., (2018) [65]	Single-blind CCT	Family therapy	23	AN	13.9 (10–17)	L-DLPFC/R-DLPFC	18 sessions; 1 mA; 20 min	*BMI***; EDI-3; EAT-26; BUT; MASC; CDI**	**−**^(c)^	Burning sensation (82%) Local redness (73%) Headache (46%) Tingling sensation (46%)
Alizadehgoradel et al., (2021) [63]	Single-blind, multi-arm RCT	Sham tDCS, MBSAT, tDCS + MBSAT	80	SUD	19.5 (18–21)	L-DLPFC/R-DLPFC	12 sessions; 1.5 mA; 20 min	*DDQ*	**X**	tDCS vs. tDCS + MBSAT vs. sham tDCS: Itching sensation (50% vs.53% vs. 38%); Burning sensation (50% vs. 59% vs. 31%); Pain (35% vs. 31% vs. 19%); Tingling (71% vs. 75% vs. 38%); Fatigue (38% vs. 29% vs. 13%); Trouble concentrating (13% vs. 12% vs. 6%)

*Italics* improvement, *Bold* no improvement, +  low risk of bias, ***X*** high risk of bias, − some concerns, *RCT* randomised controlled trial, *CCT* controlled clinical trial, *ASD* autism spectrum disorder, *ADHD* attention deficit-hyperactivity disorder, *AN* Anorexia Nervosa, *SUD* Substance Abuse Disorder, *L* Left, *R* Right, *DLPFC* Dorsolateral Prefrontal Cortex, *SOA* Supraorbital Area, *IFG* Inferior Frontal Gyrus, *IFC* Inferior Frontal Cortex, *OFC* Orbitofrontal cortex, *CT* Cognitive Training, *CRT* Cognitive Remediation Training, *MBSAT* Mindfulness-Based Substance Abuse Treatment, *ABC* Aberrant Behaviour Checklist, *RBS-R* Repetitive Behaviour Scale-Revised, *CARS* Childhood Autism Rating Scale, *CARS-GI* General Impression Item of the CARS, *CPRS-RS* Conners Parent Rating Scale-Revised-Short Form, *ERC* Emotion Regulation Checklist, *GARS* Gilliam Autism Rating Scale-Second Edition, *ToM* Theory of Mind, *CSHQ* Children’s Sleep Habits Questionnaire, *ATEC* Autism Treatment Evaluation Checklist, *CGAS* Children’s Global Assessment Scale, *CGI-I* Clinical Global Impression–Global Improvement, *DISYPS-II* Diagnostic System for Mental Disorders in Childhood and Adolescence, *VADPRS* Vanderbilt ADHD Parent Rating Scale, *CBCL* Child Behaviour Checklist, *ADHD-RS* ADHD Rating Scale, Conners 3-P Conners 3rd edition parent form, *WREMB-R* Weekly Rating of Evening and Morning Behaviour – Revised, *CIS* Columbia Impairment Scale, *ARI* Affective Reactivity Index, *MEWS* Mind Excessively Wandering Scale, *FBB* German ADHD Rating Scale, *CY-BOCS* Children’s Yale-Brown Obsessive Compulsive Scale, *OCI* Obsessive Compulsive Inventory, *BMI* Body Mass Index, *EDI-3* Eating Disorder Inventory, *EAT-26* Eating Attitudes Test, *BUT* Body Uneasiness Test, *MASC* Multidimensional Anxiety Scale for Children, Children’s Depression Inventory, *DDQ* Desire for Drug Questionnaire

^(a)^AEs assessed actively—i.e., using a questionnaire. All other controlled trials assessed AEs passively—i.e., via spontaneously reported feedback

^(b)^AEs assessed actively before and after each tDCS session with a list of side effect items (e.g., headache). Items are scored from 1 = ‘no adverse effect’ to 5 = ‘severe’. Here, the average pre-post differences in AE scores over 12 sessions are reported. Significant *p* values represent a significant decrease in AE severity over time. No significant between-group (i.e., active vs sham tDCS) differences were identified for AEs

^(c)^Moderate risk of bias assessed using ROBINS-I tool [34]. All other controlled trials were assessed using Cochrane Risk of Bias 2 (RoB 2) [33]

**Table 2:** Summary of case series and case studies using transcranial direct current stimulation in children, adolescents, and young people with psychiatric disorders

Authors (year)	Design	*N*	Diagnosis	Age: mean (range)	Anode/cathode	tDCS Protocol	Disorder-specific outcome measures	Reported AEs
D’Urso et al., (2021) [38]	Open-label, single-arm study	7	ASD	11.1 (9–13)	L-DLPFC/R-cerebellum	20 sessions; 1 mA; 20 min	*ABC-Total; ABC-social; ABC-Hyperactivity; ABC-Irritability; ABC-Stereotypic; ***ABC-Speech**	Skin irritation (43%)
Auvichayapat et al., (2020) [36]	Open-label, single-arm study	10	ASD	6.6 (5–8)	L-DLPFC/R-shoulder	5 sessions; 1 mA; 20 min	*ATEC-Total; ***ATEC-Speech**; *ATEC-Social; ATEC-Sensory; ATEC-Behaviour; CARS*	Reported there were ‘no AEs’
Wilson et al., (2018) [42]	Case study	1	ASD	18	R-TPJ/R-deltoid	8 sessions; 1.5 mA; 30 min	*ATEC-Total; ***ATEC-Speech***; ATEC-Social; ATEC-Sensory; ATEC-Behaviour*	Redness at electrode sites Ongoing tingling sensations down right arm during tDCS
Bandeira et al., (2016) [44]	Open-label, single-arm study	9	ADHD	11.1 (6–16)	L-DLPFC/R-SOA	5 sessions; 2 mA; 30 min	Only cognitive outcomes	Headache (5%) Neck pain (1%) Tingling (18%) Burning sensation (24%) Local redness (13%) Sleepiness (1%) Sense of shock (6%)
Behler et al., (2018) [57]	Case study	2	TS	19	SCM/SMA	10 sessions; 2 mA; 30 min	**YGTSS; Y-BOCS; tic count**	Headache (50%) Metallic taste during tDCS (50%)
Carvalho et al., (2015) [58]	Case study	1	TS	16	R-deltoid/SMA	10 sessions; 1.425 mA; 30 min	*YGTSS*	Not measured or reported
Shenoy et al., (2015) [54]	Case study	1	SCZ	25	L-DLPFC/L-TPJ	10 sessions; 2 mA; 20 min	*PSYRATS-AHRS*	Reported there were ‘no AEs’(^a^)
Rakesh et al., (2013) [53]	Case study	1	SCZ	24	L-DLPFC/L-TPJ	10 sessions; 2 mA; 20 min	*AHRS*	Tingling sensation Drowsiness
Andrade (2013) [51]	Case study	1	SCZ	25	L-DLPFC/L-TPP cortex	< 100 sessions; 1, 2 and 3 mA; 20–30 min	* > 90% self-reported improvement*	Tingling at electrode site
Palm et al., (2013) [52]	Case study	1	SCZ	19	L-DLPFC/R-SOA	10 sessions; 2 mA; 20 min	*PANSS; SANS*	Not measured or reported
Costanzo et al., (2015) [66]	Case study	1	Catatonia	14	L-DLPFC/R-DLPFC	28 sessions; 1 mA; 20 min	*KCRS*	Not measured or reported
Baliga et al., (2020) [59]	Case study	1	MDD	21	L-DLPFC/R-DLPFC	10 sessions; 2 mA; 30 min	*HAM-D*; **ADIS-P; ***ADIS-C*; **PARS; SCARED-C/P; CSQ-8**	Tingling sensations(^a^)
Sreeraj et al., (2016) [60]	Case study	1	MDD	23	L-DLPFC/R-DLPFC	10 sessions; 2 mA; 30 min	*HAM-A; HAM-D*	Burning sensation(^a^) Phosphenes
Sousa et al., (2021) [61]	Case study	1	SAD	24	L-vmPFC/R-vmPFC	5 sessions; 2 mA; 20 min	*LSAS-SR; SPIN*	Headache(^a^) Scalp pain Tingling Itching Burning Skin redness Drowsiness Flashes
Vaclavik et al., (2020) [62]	Open-label, single-arm study	6	Anxiety	15.2 (13–17)	L-DLPFC/R-SOA	4 sessions; 1 mA; 20 min	**ADIS-P; ***ADIS-C*;** PARS; SCARED-C/P; CSQ-8**	Headache (17%) Tingling (17%) Itching (33%) Redness (50%) Sleepiness (67%) Difficulty concentrating (17%)
Hazari et al., (2016) [55]	Case study	1	OCD	24	L-SMA/Right-SOA	28 sessions; 2 mA; 20 min	*YBOCS; HAM-A; HAM-D*	Erythematous lesion(^b^)
Narayanaswamy et al., (2015) [56]	Case study	1	OCD	24	L-SMA/R-SOA	20 sessions; 2 mA; 20 min	YBOCS; *HAM-D; HAM-A*; CGI-S(^c^)	Not measured or reported
Shariatirad et al., (2016) [64]	Case study	1	SUD	24	R-DLPFC/R-arm	24 sessions; 2 mA; 20 min	*DDQ; LDQ; CAQ; BDI*	Headache(^a^)

*Italics* Improvement, *Bold* No Improvement, *SAD* Social Anxiety Disorder, *MDD* Major Depressive Disorder, *ASD* Autism Spectrum Disorder, *SCZ* Schizophrenia, *ADHD* Attention Deficit-Hyperactivity Disorder, *TS* Tourette’s Syndrome, *OCD* Obsessive Compulsive Disorder, *SUD* Substance Abuse Disorder, *L* Left, *R* Right, *vmPFC* Ventromedial Prefrontal Cortex, *DLPFC* Dorsolateral Prefrontal Cortex, *SOA* Supraorbital Area, *TPJ* Temporoparietal Junction, *TPP* Temporoparietal, *SCM* Sternocleidomastoid Muscle, SMA Supplementary Motor Area, *LSAS-SR* Liebowitz Social Anxiety Scale Self Report, *SPIN* Social phobia Inventory, *ADIS-P* Anxiety Disorders Interview Schedule for Parents, *ADIS-C* Anxiety Disorders Interview Schedule for Children, *PARS* Paediatric Anxiety Rating Scale, *SCARED C/P* Screen for Child Anxiety Related Emotional Disorders for Children and Parents, *CSQ-8* The Client Satisfaction Questionnaire, *HAM-D* Hamilton Depression Rating Scale, *HAM-A* Hamilton Anxiety Rating Scale, *ABC* Aberrant Behaviour Checklist, *ATEC* Autism Treatment Evaluation Checklist, *CARS* Childhood Autism Rating Scale, *KCRS* Kanner Catatonia Rating Scale, *PSYRATS* The Psychotic Symptom Rating Scale, *AHRS* Auditory Hallucination Rating Scale, *PANSS* Positive and Negative Syndrome Scale, *SANS* Scale for the Assessment of Negative Symptoms, *YGTSS* Yale Global Tic Severity Scale, *Y-BOCS* Yale-Brown Obsessive Compulsive Scale, *CGI-S* Clinical Global Impressions Scale–Severity of Illness, *DDQ* Desire for Drug Questionnaire, *LDQ* Leeds Dependence Questionnaire, *CAQ* Cognitive Abilities Questionnaire, *BDI* Beck’s Depression Inventory

^(a)^AEs assessed actively—i.e., using a questionnaire. All other studies that assessed AEs collected data passively—i.e., via spontaneously reported feedback

^(b)^During the third session, the patient developed an erythematous lesion of approximately 1 cm diameter on the scalp at the site of stimulation. There was no persistent itching or pain at the site, and it resolved spontaneously

^(c)^CGI-S was included as an outcome measure but was not reported in the results

### Quality assessment

Of the 14 RCTs, overall risk-of-bias was rated as “high” in eight studies [39–41, 43, 45, 48, 63, 67], and six with “some concerns” [35, 37, 46, 47, 49, 50] (see Supplementary Material S2). The non-randomised, controlled clinical trial was rated with moderate risk of bias [65]. All open-label studies, case series, and case reports were rated as low quality.

Four RCTs were prospectively registered [40, 45, 48, 50], and three were retrospectively registered [43, 47, 63]. Among these seven protocols or trial registries, we found inconsistent and/or selective reporting in four studies. For example, one trial registered actual enrolment (i.e., updated following study completion) of 105 participants, inclusion of a wait-list control group, and a 6-month follow-up timepoint. In the final publication [40], only 41 participants were randomly assigned to receive real or sham tDCS (i.e., no wait-list control group) and there was no follow-up period. In addition, four registered primary outcomes (Early Adolescent Temperament Questionnaire; Autism Quotient (AQ); N-Back Task; and Attention Network Task) were omitted from the final publication [40], and the Cambridge Neuropsychological Test Automated Battery (CANTAB) was partially reported and relegated from a registered primary outcome to a secondary outcome. Two other studies omitted registered primary outcome measures (AQ and Gresham & Elliot Social Skills Rating Scale [43]; Dot-Probe Task and Difficulties in Emotion Regulation Scale [63]) and/or secondary outcome measures (Executive Function Checklist [43]) from final publication. Another study omitted a registered secondary outcome (Behaviour Rating Inventory of Executive Function), partially reported the Wechsler Intelligence Scale for Children, and promoted an outcome registered as “other” to a secondary outcome in the final publication (Clinical Global Impression—Severity [45]). Finally, one RCT [37] reported an incorrect clinicaltrials.gov identifier (NCT number) and we were unable to find the study record using other trial information (e.g., investigator name) in the advanced search function.

### Clinical tDCS effects in psychiatric disorders

#### Neurodevelopmental Disorders

##### Autism spectrum disorder (ASD)

Five studies applied anodal tDCS to the DLPFC in CYP with ASD. A recent multi-arm RCT [37] assigned 36 children with ASD to receive either (a) 20 sessions of anodal-tDCS to the left-DLPFC, (b) 5 sessions of anodal-tDCS to the left-DLPFC followed by 15 sessions of sham-tDCS, or (c) 20 sessions of sham-tDCS. Compared to sham, 5- and 20-sessions of anodal-tDCS were associated with significant improvements in clinician-rated total ASD severity and related symptoms, including physical health and behaviour, language, and sociability, but not sensory and cognitive awareness, at day-5, day-14, and at 6-month follow-up [37]. At 12-month follow-up, only improvements in clinician-rated total ASD severity and sociability remained significant in the 20-session tDCS group (vs. sham). Of note, no significant differences in total ASD severity or related behaviours were detected between the 5- and 20-session tDCS groups at any time point [37].

Another sham-controlled RCT [39] in 43 children with ASD reported a significant reduction in clinician-rated total ASD severity and related symptoms, including sociability, physical health and behaviour, but not sensory and cognitive awareness or language, immediately after 10-sessions of bilateral tDCS to the DLPFC (anode: left; anode: right) compared to sham. A recent single-blind RCT [41] in 40 children with ASD reported a significant improvement in sleep and observer-rated overall ASD severity immediately after 15-sessions of anodal tDCS over the left-DLPFC compared to sham, while ASD symptoms and related behaviour impairments remained unchanged [41].

The remaining two studies applied 5 sessions of anodal tDCS over the left-DLPFC: one crossover RCT in 20 children with ASD [35] reported a significant improvement in investigator- and parent-ratings of ASD symptoms, as well as parent-rated sociability, physical health and behaviour, sensory and cognitive awareness compared to sham, but not speech and language communication, at 1-week post-stimulation. Ratings of psychosocial functioning also improved at 1-week post-stimulation compared to sham, while clinical impression of improvement was rated as “much improved” in nine and “minimally improved” in eight children [35]. A single-arm open-label study [36] in 10 children with ASD reported a significant reduction in investigator-rated ASD symptoms immediately, 1 week, and 2 weeks post-treatment, compared to baseline, whereas parent-ratings of ASD symptoms relating to sociability, physical health and behaviour, sensory and cognitive awareness, but not speech and language communication, were significantly reduced at post-treatment only [36].

Only one study applied bilateral tDCS to the DLPFC (anode-left; cathode-right) in 32 children with ASD. In this double-blind RCT [43], the tDCS group showed significant improvements in (a) parent-rated emotion regulation competencies, (b) clinician-rated stereotyped behaviour, and (c) theory of mind (ToM), including first- and second-order beliefs, immediately and 1 month after 10 sessions of 1.5 mA bilateral tDCS, compared to sham. However, no significant between-group differences were detected in clinician-rated overall ASD severity and symptoms relating to communication, sociability, behavioural difficulties and precursors of ToM [43].

Two studies applied cathodal tDCS to CYP with ASD: a recent double-blind, sham-controlled RCT [40] in 41 CYP with ASD reported a significant group by time interaction in parent-rated severity of social deficits and restricted interests and repetitive behaviours due to improvement from baseline to immediately after 10 sessions of 1.5 mA tDCS to the left-DLPFC with concurrent cognitive remediation training (CRT), but not sham tDCS + CRT. A single-arm open-label study [38] applied 20 sessions of cathodal tDCS over the right cerebellar lobe in seven children with ASD, which reduced caregiver-rated aberrant behaviour symptoms 1 week after stimulation compared to baseline in all but two participants, both of whom were taking psychotropic medication during the study period. Unexpectedly, 1 week after stimulation, one patient with a history of epilepsy showed no EEG-related epileptic activity in the frontal region, while another participant with comorbid tic disorder showed fewer, less intense tics, which remained until 3-month follow-up [38].

Last, a case report [42] in an 18-year-old male with ASD applied 8 sessions of anodal tDCS to the right temporoparietal junction and reported fewer total ASD symptoms immediately and 2-months after stimulation compared to baseline.

##### Attention deficit hyperactivity disorder (ADHD)

Two double-blind RCTs applied tDCS to the inferior frontal cortex (IFC). The largest RCT [50] in CYP with ADHD (*n* = 50) administered 15 sessions of anodal or sham tDCS to the right-IFC with concurrent cognitive training (CT). Compared to sham, the tDCS group showed significantly higher parent-rated ADHD symptoms immediately after stimulation, but not at a 6 months’ follow-up. No significant differences were reported in other measures of ADHD symptoms or related impairments [50]. The other RCT [46] administered five sessions of bilateral high-definition tDCS to the right- and left-IFC in 33 children and adolescents with ADHD, with stimulation intensity titrated post-randomisation to 0.25 mA (*n* = 11) and 0.5 mA (*n* = 9) to minimise discomfort. Results showed a significant reduction in self-rated ADHD total and hyperactivity symptoms compared to sham at post-treatment, but not at 4-month follow-up, while self-rated inattention or impulsivity and all parent-rated ADHD symptoms remained unchanged [46].

Four studies applied anodal tDCS to the left-DLPFC: a recent double-blind, sham-controlled RCT [48] with 25 CYP with ADHD reported no significant between-group differences in parent-rated severity of ADHD symptoms or related behaviours immediately after 12 sessions of 1 mA tDCS with concurrent CT, compared to sham tDCS + CT. A smaller double-blind, crossover RCT [49] in 15 adolescents with ADHD reported significant improvement in parent-rated inattention at 1-week follow-up, but not immediately after five sessions of tDCS, compared to sham. No other clinical effects were found [49]. Another double-blind, crossover RCT [45] in 19 children with ADHD compared five sessions of transcranial random noise stimulation (tRNS) over the left-DLPFC and right-IFC with tDCS to the left-DLPFC, both with concurrent executive function training. Findings showed that relative to tDCS, tRNS significantly reduced parent-rated ADHD symptoms immediately and 1 week after stimulation (adjusting for baseline scores), but with no difference in global clinical impressions [45].

Last, in a single-arm open-label study [44] in nine children with ADHD, five sessions of anodal tDCS over the left-DLPFC were combined with a picture association cognitive training task and parents reported overall improvements in behaviour. However, without a sham-control, a placebo effect cannot be ruled out.

##### ASD and ADHD

A double-blind, parallel, sham-controlled case report [67] applied 10-sessions of anodal-tDCS over the right inferior frontal gyrus (rIFG) combined with cognitive training in two 15-year-old, female, fraternal adolescent twins with ASD, ADHD, and anxiety. Both twins had parent-reported compulsive symptoms, and one had comorbid OCD. Compared to baseline, findings showed reduced parent-rated compulsive and repetitive/restrictive symptoms, but not clinician-rated OCD symptoms or parent-rated ADHD symptoms, immediately after anodal-tDCS. No clinical changes were observed following sham-tDCS [67].

##### Tourette’s syndrome

In one case study [57] in an 18-year-old female and 20-year-old male with motor and vocal tics, bilateral cathodal tDCS was applied to the right- and left-pre-supplementary motor area (SMA) twice-daily over 5 consecutive days. Compared to baseline, one participant showed a reduction in tics immediately after stimulation, whilst the other participant showed an increase in tics and OCD symptoms. Both participants’ self-rated negative affect decreased, and positive affect increased, but none of these changes were statistically tested [57]. One case study in a 16-year-old boy with refractory Tourette’s syndrome showed reduced motor and vocal tics immediately, 3-, and 6-weeks after ten sessions of cathodal tDCS over the left pre-SMA compared to baseline [58].

#### Schizophrenia-spectrum disorders

##### Schizophrenia

Three case studies applied bilateral tDCS (anode-left-DLPFC; cathode-right-temporo-parietal junction) over 5 consecutive days. In two of these studies, twice-daily tDCS reduced auditory hallucinations entirely at post-treatment in a 24-year-old male [53] and almost entirely 1-month after stimulation in a 25-year-old pregnant female [54], whereas in the third study [51], once-daily tDCS reduced auditory hallucinations immediately after stimulation in a 25-year-old female, but she required continued once- or twice-daily stimulation to maintain improvements over 3 years. Last, one case study in a 19-year-old-male reported reduced positive and negative symptoms, disorganization, flattened affect, lack of concentration, and impetus after ten sessions of anodal-tDCS over the left-DLPFC [52].

##### Catatonia

A case study [66] in a 14-year-old female with ASD and catatonia showed reduced catatonic symptoms compared to baseline immediately and 1-month after 28-sessions of bilateral tDCS (anode-right; cathode-left) over the DLPFC.

#### Major depressive disorder

Two case studies applied ten sessions of bilateral tDCS over the left- and right-DLPFC. One study [59] reported that a 21-year-old male presenting with a moderate depressive episode showed fewer depressive symptoms immediately after 5-sessions of stimulation applied on two occasions separated by 2 years. The second [60] reported that a 23-year-old pregnant female with a depressive episode showed fewer depressive and anxiety symptoms 1 month after stimulation compared to baseline, which, the authors suggested was indicative of clinical remission.

#### Anxiety disorders

A single-arm, open-label study [62] in six adolescents with social anxiety disorder or generalized anxiety disorder reported that combined anodal-tDCS to the left-DLPFC with attention bias modification training reduced self- and parent-reported total anxiety symptoms, self- and parent-rated anxiety-related emotional symptoms, and clinician-rated anxiety symptoms, compared to baseline. However, only self-reported total anxiety symptoms significantly reduced from baseline to post-stimulation [62].

A case study [61] in a 24-year-old female with social anxiety disorder reported reduced self-reported social anxiety symptoms immediately after five sessions of anodal tDCS over the left vmPFC, and at 15 days’ follow-up, although improvements did not reach clinical significance.

#### Obsessive compulsive disorder (OCD)

Two case studies applied anodal tDCS over the left pre-SMA/SMA twice-daily over 10 conseuctive days. In one [55], a 24-year-old male with OCD and co-occurring depressive symptoms showed fewer OCD symptoms compared to baseline immediately and 7 months after stimulation. The same stimulation protocol was applied for eight sessions 2 years later, which reduced recurring OCD and depressive symptoms, but also lesioned the skin under the stimulation site [55]. In the second case study [56], a 24-year-old male showed a reduction in OCD, depression, and anxiety symptoms immediately, 1 week and 1 month after stimulation.

#### Substance abuse disorder

A single-blind, parallel group RCT [63] in 80 boys with methamphetamine addiction administered 12-sessions of anodal tDCS over the left-DLPFC with or without combined mindfulness training, mindfulness training only and sham tDCS only. The results showed a significant reduction in desire for drugs at post-treatment and 1-month follow-up in the three active treatment groups, but not in the sham group, compared to baseline [63].

In a case report [64] in a 24-year-old male with methamphetamine use disorder, anodal-tDCS over the right DLPFC reduced self-reported drug cravings immediately after 20 sessions of stimulation and, after four more sessions, at 6 months’ follow-up, at which point paranoid delusions and hallucinations had also reduced completely.

#### Eating disorders

One clinically controlled trial [65] in 23 adolescents with anorexia nervosa (AN) combined treatment as usual with either family therapy or 18-sessions of bilateral tDCS over the DLPFC (anode-left; cathode-right). It was not clear how people were allocated to these two study arms. Compared to baseline, only the tDCS group had significantly reduced BMI immediately after stimulation and at 1-month follow-up. Both groups showed significantly reduced overall AN, depression, and anxiety symptoms compared to baseline, but no significant Group by Time interaction, and thus, placebo effects cannot be ruled out [65].

### Mood tDCS effects in psychiatric disorders

#### ADHD

A double-blind, sham-controlled RCT [48] reported no significant differences in parent-rated mood or anxiety scores following 12 sessions of anodal-tDCS to the left-DLPFC with cognitive training (CT) compared to sham + CT.

#### OCD

Two case studies [55, 56] reported reduced clinician-rated depression symptoms on the Hamilton Depression Rating Scale following anodal-tDCS to the SMA compared to baseline.

#### Substance abuse disorder

In a case report [64], the participant self-reported reduced depression, on the Beck Depression Inventory immediately, 3-months, and 6-months after anodal-tDCS to the right-DLPFC compared to baseline.

### Neurocognitive tDCS effects in psychiatric disorders

#### ASD

A double-blind RCT [40] reported that improvements in parent-reported social communication and reduced restricted, repetitive behaviours were significantly associated with improved emotion recognition (CANTAB Emotion Recognition Task) and cognitive flexibility (composite score of: (1) time taken to complete the Color Trail Test 2, (2) switch cost in the CANTAB Multitasking Test, and (3) mean reaction time during the WCST rule-switching block) following cathodal tDCS to the left-DLPFC + CRT, compared to sham + CRT. In addition, results showed significant improvements in information processing (composite score of: (1) time taken to complete the Color Trail Test 1, and (2) CANTAB Reaction Time Test) following cathodal-tDCS + CRT, compared to sham [40].

#### ADHD

A recent RCT [48] reported no significant between-group differences in neurocognitive performance on the CANTAB after 12-sessions of 1 mA tDCS to the left-DLPFC combined with cognitive training (CT), compared to sham tDCS + CT. Another recent sham-controlled, crossover study [47] in 11 CYP with ADHD reported a significant reduction in number of omission errors (i.e., inhibitory control) in the real tDCS group, compared to sham, immediately after receiving 5 sessions of 1.5 mA cathodal tDCS to the left-DLPFC, but not at 1-week or 1-month follow-up. No significant between-group differences were detected for auditory continuous performance (i.e., sustained attention) immediately, 1-week, or 1-month after real tDCS, compared to sham tDCS [47].

A double-blind RCT [46] comparing 0.5 mA and 0.25 mA to sham reported a significant reduction in reaction time variability in a combined Go/No-Go and n-back task immediately and 4-months after 5-sessions of 0.5 mA anodal HD-tDCS over the right IFC. In contrast, in the same task, the 0.25 mA group showed an increase in no-go commission errors over the course of tDCS, and this effect became significant at day-5, but was non-significant at post-stimulation and at 4-month follow-up. At post-stimulation, the 0.5 mA group also showed a significant reduction in reaction time variability in the flanker task and a reduced number of commission errors in the spanboard task compared to sham, but neither effect was significant at follow-up [46].

One double-blind, crossover trial [49] showed that compared to sham, anodal tDCS improved (a) QbTest (a combined working memory (n-back minus-2) and go/no-go task) measures of attention at 7-day follow-up only and, (b) measures of hyperactivity immediately and 7-days after stimulation, but not measures of impulsiveness. In a double-blind, crossover RCT [45], tRNS improved working memory, but not short-term memory, and only processing speed in a sustained attention task, compared to tDCS. In addition, exploratory moderation analysis predicted a trend-level larger tRNS effect in parent-rated ADHD symptoms for participants with the greatest working memory improvement.

In a double-blind, parallel RCT [50], there were no significant effects of anodal-tDCS to the right-IFC across measures of motor and interference inhibition, time estimation, sustained attention, cognitive flexibility, visuospatial working memory, and three task-independent measures processing speed, intrasubject response variability, and prematurity, compared to sham. Finally, a single-arm open-label study [44] reported a significant reduction in errors on attention (omission) and switch tasks after anodal-tDCS to the left-DLPFC compared to baseline, but no improvement in verbal or visuospatial working memory.

#### Schizophrenia

One case study [52] reported faster completion time on the Trail Making Test (TMT) Part A and B and fewer errors on the Self-Ordered Pointing Task (SOPT) one- and two-weeks after anodal-tDCS to the left-DLPFC compared to baseline.

#### Substance abuse disorder

A single-blind, parallel RCT [63] reported a significant group by time interaction in n-back task reaction times and accuracy, Wisconsin Card Sorting Task perseverative errors, and in a risk-taking task, all due to an improvement from baseline to immediately and 1-month after (a) tDCS only, (b) tDCS + mindfulness training, or (c) mindfulness training only, but not sham. However, there were no equivalent effects on any of the go/no-go task measures.

A case study [64] reported consistent improvement on subscales of the Cognitive Abilities Questionnaire that measured memory, inhibitory control, selective attention, decision making, planning, sustaining attention, and cognitive flexibility, but not social cognition, from baseline to 2-months, 4-months, and 6-months after anodal-tDCS to the right-DLPFC.

##### Safety

Overall, tDCS was well-tolerated and feasible in a variety of age groups and psychiatric disorders, which extends existing evidence of a good side-effect and tolerability profile of tDCS in children and adolescents [20]. However, adverse events (AEs) were not measured or reported in six studies [43, 47, 52, 56, 58, 66] and whilst four studies reported no AEs, it was not clear whether sensory side effects (e.g., tingling sensation) were measured [35, 36, 39, 54]. Only 10 studies reported monitoring AEs actively (i.e., using a structured questionnaire that lists specific AEs), whereas the remaining 16 studies monitored AEs passively and often relied on spontaneous feedback from participants or caregivers (see Table 1 & 2). Here, a selective reporting bias is very likely as the frequency of AEs reported increases when monitored actively [68]. Future studies should collect data for AEs actively, using a structured questionnaire (e.g., [68]) in which the rater asks for each specific AE (e.g., headache or itching).

Across studies, one severe adverse event (SAE) was reported, which was an erythematous lesion. The lesion was ~ 1 cm in diameter at the site of stimulation and developed during the third session of the patient’s second course of tDCS (patient had received 20 tDCS sessions 7-months prior) [49]. The authors noted that the lesion was not experienced as itchy or painful, and that it resolved spontaneously. Skin lesions and/or thermic damage appear to be rare and likely result from improper tDCS preparation or administration (e.g., poor electrode skin contact from dry sponges) [69, 70]. Therefore, it is imperative that the condition of tDCS electrodes is closely monitored over time and that care is taken when administering saline to the sponge of electrodes to prevent tDCS-related skin damage.

### Unpublished registered trials

Of the 11 registered trials (see Table 3), four have not started recruiting, one has been completed (October 2021), and the remaining six are ongoing. Three are quadruple-blind RCTs, four are triple-blind (one crossover; three RCTs), two are double-blind RCTs, two are open label (one single-arm; one-RCT). These 11 studies are (a) recruiting either ASD (*n* = 6), ADHD (*n* = 2), or MDD (*n* = 3); (b) stimulating the DLPFC (*n* = 7), temporal parietal junction (*n* = 1), or using a neuroimaging biomarker (*n* = 1); (c) applying tDCS alone (*n* = 2), or combining stimulation with cognitive training (*n* = 4), mindful breathing training (*n* = 1), applied behaviour therapy (*n* = 1) or medication (*n* = 3; and (e) recruiting ~ 80 participants (range: 15–172) per trial, in CYP aged (on average) between 10 and 18 years old.

**Table 3:** Summary of ongoing unpublished clinical trials using transcranial direct current stimulation in children, adolescents, and young people with psychiatric disorders

Trial ID (status)	Design	*N*^(a)^	Diagnosis	Age range	Control	Anode/cathode	Protocol	POM
NCT05492032 (not yet recruiting)	Triple-blind, crossover RCT	90	ASD	14–21	Sham tDCS	DLPFC^(b)^	10 sessions; 1 mA; 20 min combined with CT	SRS-2; CANTAB
NCT045491720 (not yet recruiting)	Triple-blind RCT	45	ASD	4–17	1 mg Risperidone/Sham tDCS + placebo tablet	L-DLPFC/R-SOA	10 sessions; 1.5 mA; 20 min; combined with drug placebo	GADS; Verbal Fluency Task; ToMT
NCT05035511 (recruiting)	Open-label, single-arm study	90	ASD	16–22	None	n/r	10 sessions; n/r; 20 min combined with CT	SRS-2
NCT05105126 (not yet recruiting)	Quadruple-blind RCT	24	ASD	5–12	Sham tDCS	L-DLPFC/R-DLPFC	20 sessions; 1 mA; 20 min combined with applied behavior analysis	BRIEF, EEG
Luckhardt et al. (2021) [71]	Double-blind RCT	100	ASD	10–17	Sham tDCS	L & R-TPJ^(f)^	10 sessions; 2 mA; 20 min combined with CT	SRS
Prillinger et al. (2021) [72]	Double-blind RCT	20	ASD	12–17	Sham tDCS	L-DLPFC/R-SOA	10 sessions; 2 mA; 20 min	SRS
NCT04704687 (Recruiting)	Open-label RCT	150	ADHD	7–14	Sham tDCS	n/r	15 sessions; n/r; n/r combined with CT	ADHD-RS
Guimarães et al. (2020) [73]	Triple-blind RCT	15	ADHD	6–16	Sham tDCS	L-DLPFC/R-SOA	5 sessions; 2 mA; 30 min	TAVIS
NCT05498441 (not yet recruiting)	Triple-blind RCT	120	MDD	13–18	Routine HD-tDCS + quetiapine, lithium and/or divalproate	Neuroimaging biomarker^(c)^	20 sessions; 2 mA; 20 min	HAMD-17; RBANS; ALFF
NCT04780152 (recruiting)	Quadruple-blind RCT	172	MDD	10–17	Sham tDCS + fluoxetine	L-DLPFC/ R-DLPFC	10 sessions; 2 mA; 30 min	CDI
NCT03897699 (completed^(d)^)	Quadruple-blind RCT	68	MDD	16–24	Sham tDCS	DLPFC^(b)^	2 mA; 20 min combined with mindful breathing training^(e)^	fMRI

*MDD* Major Depressive Disorder, *ADHD* Attention Deficit-Hyperactivity Disorder, *ASD* Autism Spectrum Disorder, *L* Left, *R* Right, *DLPFC* Dorsolateral Prefrontal Cortex, *SOA* Supraorbital area, *TPJ* Temporoparietal Junction, *HD-tDCS* High-Definition tDCS, *GADS* Gilliam Autism Rating Scale, *ToMT* Theory of Mind Test, *CDI* Childhood Depression Inventory, *ADHD-RS* ADHD Rating Scale, *DBD-RS* Disruptive Behaviour Disorders Rating Scale, *VAS* Visual Analogue Scale, *GNG* Go/No-Go, *SRS-2* Social Responsiveness Scale-2nd Edition, *CANTAB* Cambridge Neuropsychological Test Automated Battery, *ERP* Event-Related Potential, *EATQ-R-EC* Early Adolescent Temperament Questionnaire—Revised—Effortful Control Subscale, *AQ* Autism Quotient, SRS Social Responsiveness Scale, *RTA* CANTAB Reaction Time, *OTS* One Touch Stockings of Cambridge, *MTT* Multitasking Test, *ERT* Emotion Recognition Task, *ANT* Attention Network Task, *EEG* Electroencephalogram, *fNIRS* Functional Near-Infrared Spectroscopy, *BRIEF* Behavior Rating Inventory of Executive Function, *fMRI* Functional Magnetic Resonance Imaging, *HAMD-17* Hamilton Depression Rating Scale (17 items), *RBANS* Repeatable Battery for the Assessment of Neuropsychological Status, *ALFF* Amplitude of Low-Frequency Fluctuation (fMRI), *TAVIS* test of visual attention

^(a)^Anticipated

^(b)^tDCS montage (anodal, cathodal, or bilateral) not reported

^(c)^The stimulation target and electrode polarity will be based on neuroimaging biomarkers extracted via machine learning. Sham tDCS group receives 20 sessions of “routine” (i.e., non-personalised) anodal HD-tDCS to the left-DLPFC at 2 mA for 20 min

^(d)^October 2021

^(e)^Number of tDCS sessions not reported

^(f)^HD-tDCS, with several cathode electrodes surrounding each anode

## Discussion

This is the first systematic review that collates published and unpublished studies investigating the effects of multi-session tDCS applied to CYP with psychiatric disorders. To date, studies are limited to case studies/series (*n* = 14), open-label single-arm studies (*n* = 4), sham- or active-controlled trials with < 50 participants (*n* = 13) or > 50 participants (*n* = 2). These studies demonstrate tDCS is well-tolerated, and that it is feasible to conduct RCTs in CYP, particularly those with ADHD and ASD. There is some encouraging evidence of improvement in clinical, cognitive, or mood measures, however, it is not possible to determine the therapeutic efficacy of multi-session tDCS for CYP with psychiatric disorders.

Of the 33 included studies, 30 measured clinical effects immediately after the final tDCS session, with all except six [45, 46, 48–50, 57] reporting an improvement in at least one outcome measure of core disorder-specific symptoms. Of the 19 studies that measured clinical effects at a longer-term follow-up, improvements in core symptoms persisted at 1-week [36, 56], 2-weeks [36, 37, 61], 3-weeks [58], 1-month [43, 54, 56, 60, 63, 65, 66], 6-weeks [58] 6-months [37, 64], 7-months [55], or 12-months [37] after the final session of tDCS, with one study reporting no effect at 4-months [46]. Overall, these findings are in line with evidence of improvement in clinical outcomes in adults with psychiatric disorders, which have been shown to persist up to at least one-month post-stimulation (e.g. [29]). Interestingly, clinical effects only persisted on a once- to twice-daily tDCS maintenance regime in one case [51] or were significantly improved at 1-week but not immediately after stimulation [49]. This might relate to findings showing delayed tDCS effects, such that improvements only emerge after the acute treatment phase [73]. However, the evidence of carryover effects in Soff et al., [49] limited their analyses to the first phase of the crossover study with a very small sample size (N = 15). Therefore, larger parallel-group RCTs are needed to examine changes in core and related symptoms of psychiatric disorders in CYP immediately after tDCS as well as at one or multiple longer-term follow-ups.

Relatively few studies measured neurocognitive or mood outcomes. 11 studies measured neurocognitive outcomes, of which seven found tDCS-related improvements in the first assessment immediately [40, 44, 46, 47, 63], 1-week [52], or 1-month [64] after the last stimulation session, which persisted in five of the studies until 1-week [49], 2-weeks [52], 1-month [63], 4-months [46], and 6-months [64] follow-up. Three studies reported no effect of tDCS on cognition compared to sham [48, 50] or tRNS [45] at post-stimulation and/or follow-up. Three of the four studies measuring mood outcomes found improvements immediately after tDCS [55, 56, 64], with one study testing and finding the effect at 3- and 6-months follow-up [64]. One study reported no effect of tDCS on mood compared to sham [48].

In the 13 studies with cognitive and/or mood outcomes, all except four [46, 50, 55, 56] stimulated the DLPFC. This is in line with substantial meta-analytic evidence that the DLPFC subserves executive functions or regulates mood [e.g. [74–77].), and with tDCS studies in healthy controls, which have reported improved cognitive outcomes up to 12-months [78] and in mood outcomes [79] post-stimulation. Twelve other studies also stimulated the DLFPC, but none measured cognitive or mood outcomes. Impaired executive functioning and mood regulation mediate the pathophysiology of many psychiatric disorders (depression [80], schizophrenia [81], ADHD [82], and ASD [83]). Further, patients often desire alternative treatments that improve executive functioning or mood over symptoms [84] without side effects associated with pharmacological interventions, such as secondary blunted affect [85], weight gain and poor social functioning [86]. It is therefore important that future research measure the effects of tDCS on a variety of disorder-relevant cognitive outcomes and mood impairments.

Heterogeneous stimulation protocols and lack of dosage-guidance meant we were not able to identify optimal stimulation parameters in CYP with psychiatric disorders. This is of concern given the neurophysiological effects of tDCS may be non-linear, and because emerging evidence suggests tDCS may modulate cortical excitability via the scalp and/or peripheral nerves, which may complicate predictions about the dose–response relationship and the reproducibility of findings [87]. For example, in one RCT [46], adolescents with ADHD received sham (*n* = 13), or 0.5 mA (*n* = 9), or 0.25 mA (*n* = 11) anodal HD-tDCS to the IFC depending on cutaneous sensitivity. Compared to sham, the 0.25 mA group showed significantly reduced response inhibition, an effect not observed in the 0.5 mA group, which opposed the authors hypothesis that increasing right-IFC activity would improve executive function. In addition, in one case series [57], cathodal tDCS to the motor cortex did not improve symptoms in Tourette’s syndrome, and actually increased tic-count, whereas in another case report [58], cathodal tDCS to the pre-SMA improved both motor and verbal tics.

The majority of studies (*n* = 30) used a stimulation intensity of 1–2 mA, based on studies in adult populations. However, the stimulation intensity required to modulate cortical excitability in a polarity-dependent manner or induce longer-term effects that persist after stimulation cessation in CYP may differ from that in adults [88]. The issue of safety in tDCS in CYP is often regarded as not being a major concern given that both the type and the magnitude of adverse effects do not differ between CYP and adults. However, it is of note that various anatomical parameters change with age (e.g., scalp-to-brain distance) and evidence shows that in CYP, lower current intensities (e.g., 1 mA) can achieve brain current densities seen in adults at 2 mA current [88, 89]. Application of tDCS on the basis of parameters used in adults may, therefore, produce larger and potentially unintended or adverse effects in CYP. An example of unanticipated findings (albeit positive, rather than adverse) was reported in D’Urso et al.’s [38] study of cathodal tDCS applied to the cerebellum of CYP with ASD. It reported the remission of two frontal epileptic foci in one participant who had lifelong comorbid epilepsy and in another participant who had comorbid tic disorder, there was a 90% improvement in frequency and intensity of tics, and this was maintained until 3-month follow-up. This could be evidence of indirect stimulation of non-target sites [90], leading to possible unintended modulation of symptoms, behaviour or cognition in a potentially clinically meaningful manner. This is relevant in the wider ethical debate surrounding direct-to-consumer marketing of tDCS devices sold for non-clinical or “neuroenhancement” purposes (see [91] for review) and is especially relevant in the context of CYP with psychiatric disorders (see [92] for review).

Overall, inconsistent or unexpected findings (i.e. [38, 46, 57, 58]) underline the need to improve understand the underlying biophysiological mechanisms of tDCS, as well as how different parameters (e.g., stimulation intensity) interact with the stimulated tissue of a developing brain. One way to address this and identify optimal parameters is for future studies to broaden outcome measures to capture potential unintended effects on regions functionally related to target areas, and to explore the parameter space ideally using Bayesian optimisation (e.g. [93]), focal forms of tDCS (e.g., HD-TDCS), or open-source computational modelling software (e.g., ROAST; [94]).

## Limitations

Interpretation is constrained by methodological limitations present in included studies. Heterogeneity in study design, outcome measures, stimulation protocols, and participant characteristics (e.g., age, gender, disorder profile) limited comparisons across findings and adequately powered meta-analyses of clinical, cognitive, or mood outcomes. All open-label studies and case reports or series were rated as poor quality, while RCTs had some concerns (*n* = 6) or a high (*n* = 7) risk of bias. These ratings were mainly due to a lack of detail regarding randomisation and allocation concealment. Only 17 studies performed any statistical analysis on outcome measures, and of those studies that reported statistically significant effects, four were open-label or case series/reports. Across studies, one [50] corrected for multiple testing and three [40, 48, 50] assessed integrity of blinding of parents, raters and/or experimenters, i.e., it cannot be ruled out that effects were due to placebo or test–retest effects, false-positives, and/or bias by knowledge of group assignment [95]. Only eight studies combined stimulation with cognitive training, which has been reported to boost and prolong the effects of tDCS [96, 97]. Sample sizes were between 15 and 50, which is short of that required to detect a medium effect in cognitive tasks (e.g. [98]).

It appears that the direction of traffic is towards improving the quality of studies. This is reflected by the fact that the majority of the 11 ongoing, or upcoming, trials we identified are double-, triple-, or quadruple-blind RCTs with larger sample sizes (~ 70 on average) with half combining tDCS with cognitive training across multiple sessions. However, 8 out of 11 registered trials are recruiting children with either ASD or ADHD; thus we cannot be sure that the same improvement in study quality will be seen across other psychiatric disorders or non-registered trials.

## Conclusion

Although encouraging, the evidence to date is insufficient to conclude that tDCS can improve clinical symptoms, mood, or cognition in CYP with psychiatric disorders. This is largely due to the heterogeneous study designs, limited outcomes, and small sample sizes, as these limit the interpretability and comparability of findings across studies. Future studies should seek to confirm existing findings with larger samples, and randomised, sham-controlled designs that include measures of clinical, cognitive, and mood outcomes immediately after stimulation and in longer-term follow-ups. Stimulation protocols should be justified and should consider any possible unintended outcomes that might occur, particularly in younger populations.

## Supplementary Information

Below is the link to the electronic supplementary material.

Supplementary file1 (DOCX 560 KB)

## Data Availability

Data sharing is not applicable to this article as no datasets were generated or analysed during the current study.

## Declarations

### Permission

We have not used figures from other sources or that have been published elsewhere.
