# Supplementary material for: Effects of transcranial direct current stimulation in children and young people with psychiatric disorders: a systematic review
Source: Eur Child Adolesc Psychiatry. 2023 Feb 11;33(9):3003–23. doi: 10.1007/s00787-023-02157-0 (PMC11424672; doi:10.1007/s00787-023-02157-0)
Supplement: Supplementary file 1 — Supplementary file1 (DOCX 560 KB) [file 787_2023_2157_MOESM1_ESM.docx]

*Supplementary Material S1: Data extraction*

We extracted the following information: (a) study characteristics (e.g. article title, reference, study design, sample size calculation); (b) participants (e.g. sample size, age, gender, inclusion/exclusion criteria, main disorder, illness severity, comorbidities); (c) tDCS (type of tDCS used (i.e. anodal, cathodal), site of stimulation, stimulation intensity, duration, total number of sessions); (d) comparators (sham, treatment as usual, waitlist, no comparison); (e) concurrent treatment (medication, psychotherapy, cognitive remediation); (f) outcomes (disorder-specific symptoms, mood, cognition, adverse effects).

*
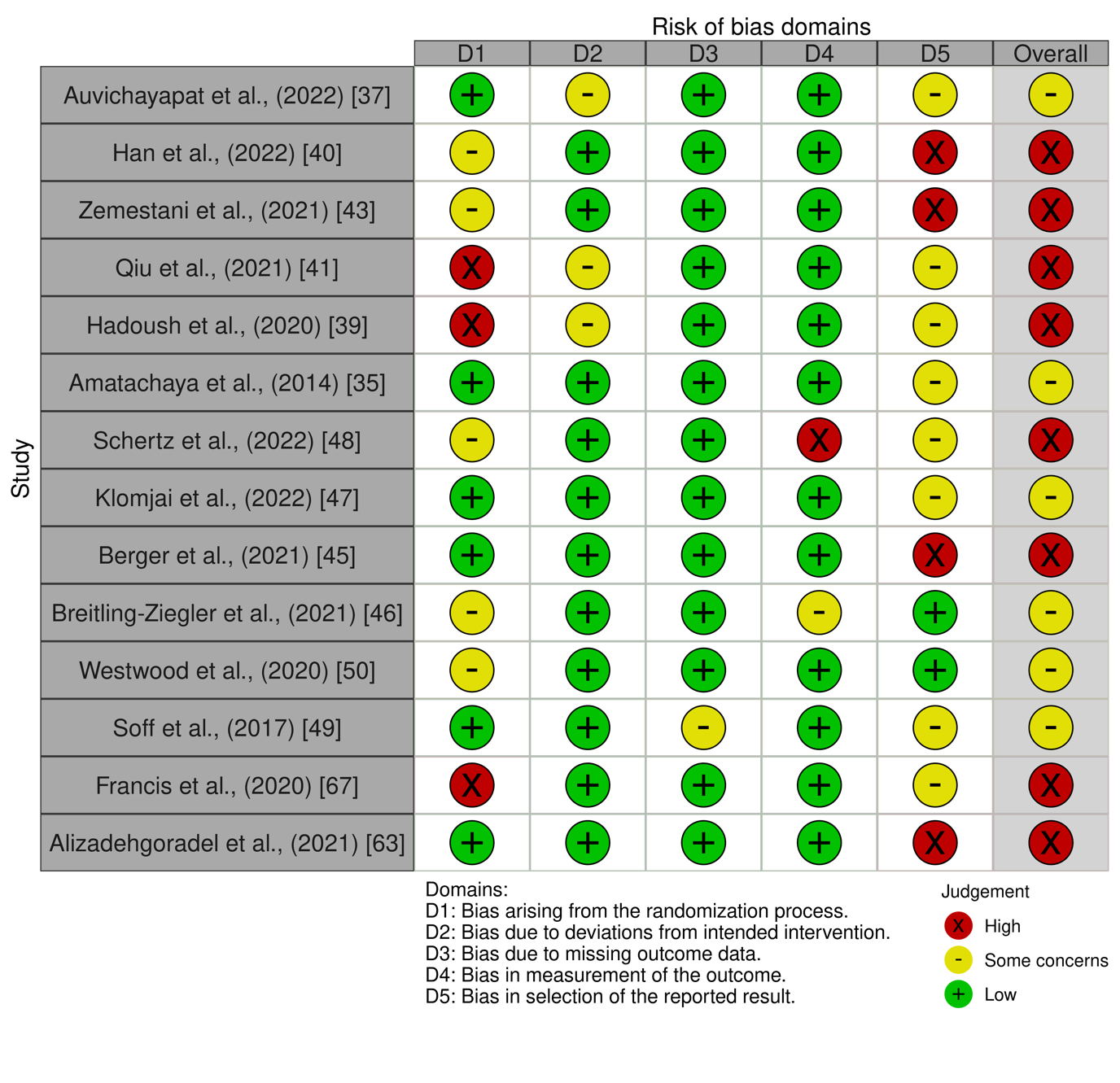
Supplementary Material S2: Results of risk of bias assessment*

***
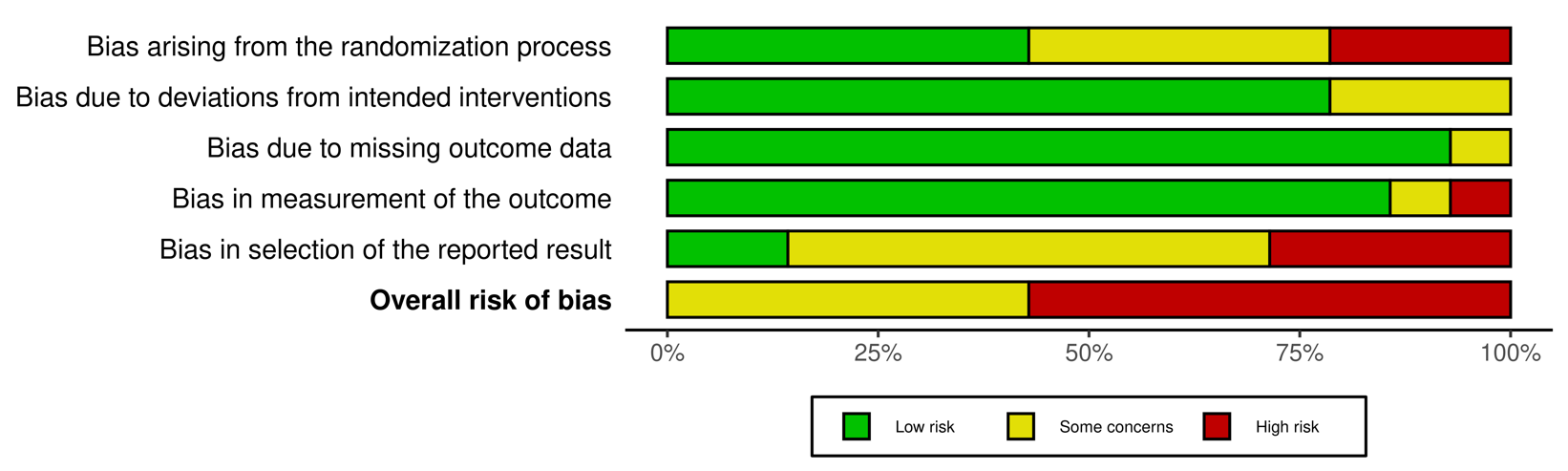
***
